# Supplementary material for: Invasive Salmonella exploits divergent immune evasion strategies in infected and bystander dendritic cell subsets
Source: Nat Commun. 2018 Nov 19;9:4883. doi: 10.1038/s41467-018-07329-0 (PMC6242960; doi:10.1038/s41467-018-07329-0)
Supplement: Supplementary file 1 — Supplementary Information [file 41467_2018_7329_MOESM1_ESM.pdf]

Supplementary Information for:

Invasive *Salmonella* Exploits Divergent Immune Evasion Strategies in Infected and Bystander Dendritic Cell Subsets

Aulicino & Rue-Albrecht et al.

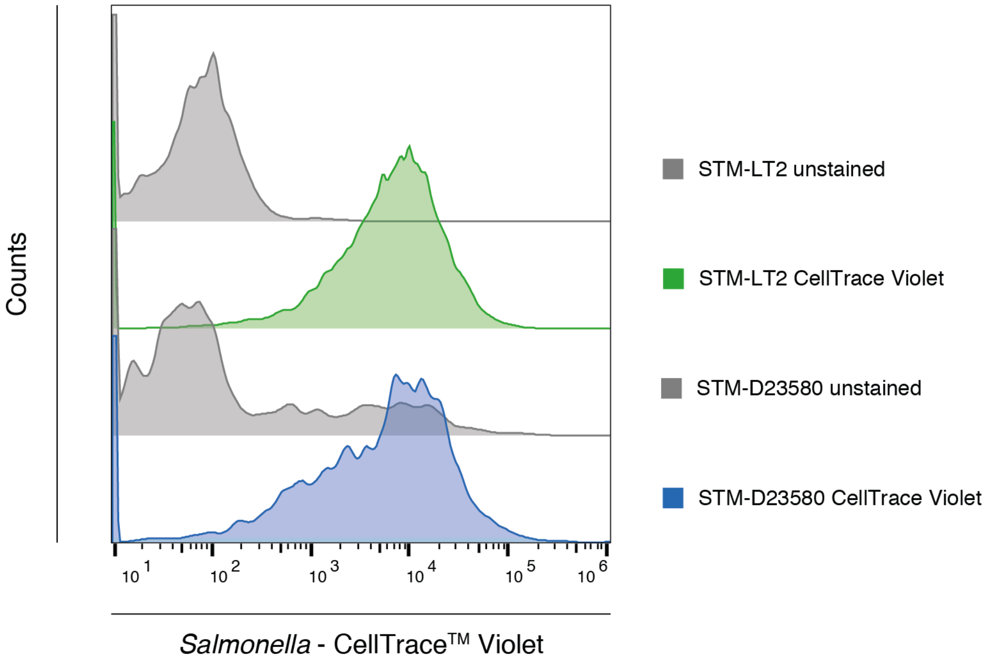

Supplementary Figure 1. Bacterial staining with CellTrace ™ Violet dye.

Histogram representing the CellTrace ™ Violet fluorescent intensities of STM-D23580 (blue) and STM-LT2 (green). Unstained bacteria are represented by the grey curve.

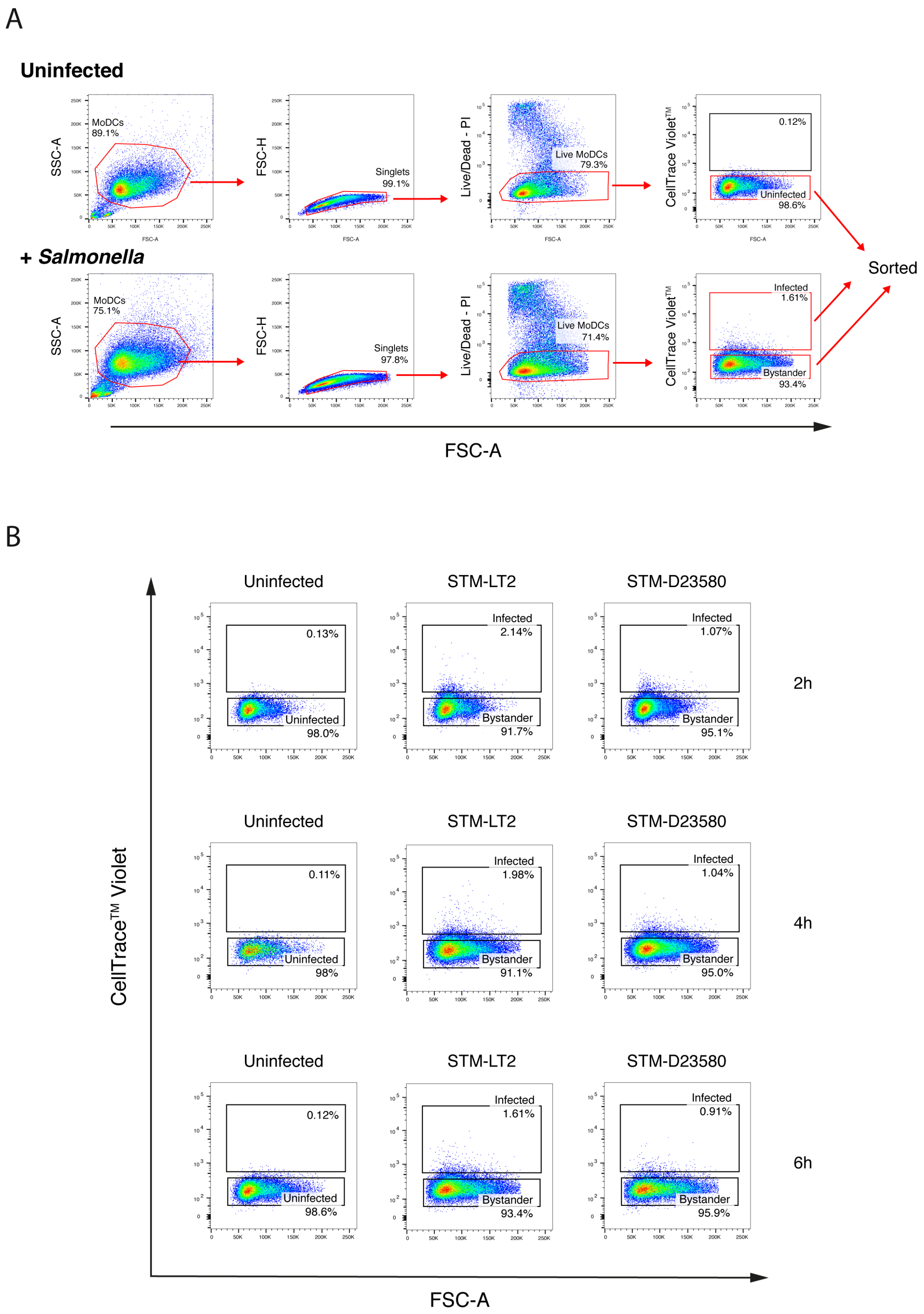
A
Uninfected
+ Salmonella
SSC-A
FSC-H
Live/Dead - PI
CellTrace Violet™
MoDCs
89.1%
Singlets
99.1%
Live MoDCs
79.3%
0.12%
Uninfected
98.6%
MoDCs
75.1%
Singlets
97.8%
Live MoDCs
71.4%
Infected
1.61%
Bystander
93.4%
Sorted
FSC-A
B
Uninfected
STM-LT2
STM-D23580
0.13%
Uninfected
98.0%
Infected
2.14%
Bystander
91.7%
Infected
1.07%
Bystander
95.1%
2h
0.11%
Uninfected
98%
Infected
1.98%
Bystander
91.1%
Infected
1.04%
Bystander
95.0%
4h
0.12%
Uninfected
98.6%
Infected
1.61%
Bystander
93.4%
Infected
0.91%
Bystander
95.9%
6h
CellTrace™ Violet
FSC-A

Supplementary Figure 2. FACS plots of uninfected and *Salmonella*-challenged MoDCs over the course of the infection.

A Representative example of the gating strategy used to sort uninfected, bystander, or infected MoDCs. **B** Representative time-course of FACS scatter plots of MoDCs challenged with STM-LT2 or STM-D23580 or left uninfected. The upper gate displaying a high CellTrace™ Violet fluorescence intensity captures MoDCs that engulfed *Salmonella*, (Infected), while the lower gate (Bystander) includes the MoDCs that did not exhibit violet fluorescence.

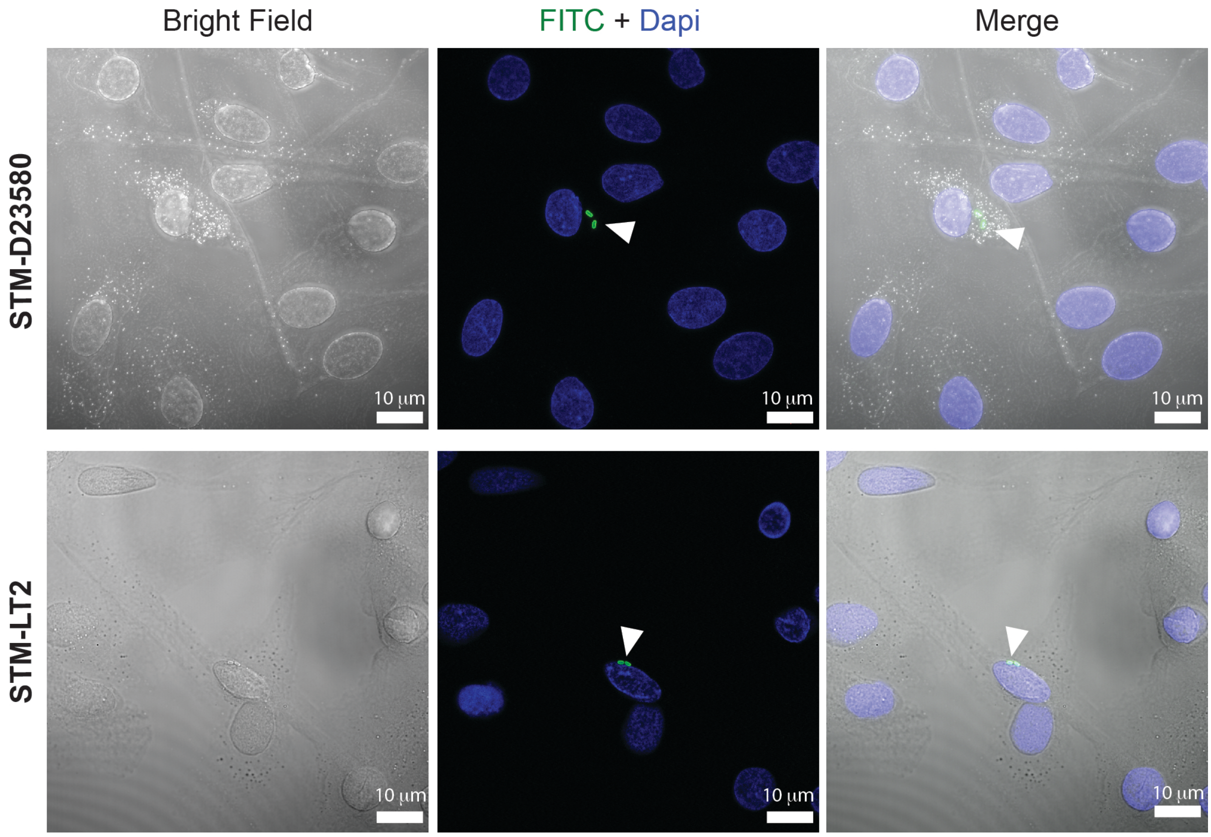

Supplementary Figure 3. Uptake of bacteria by MoDCs.

Representative images of infected MoDCs containing STM-LT2 or STM-D23580 using bright field and fluorescence microscopy are shown. Nuclei were stained with DAPI (blue) and *Salmonella* (green) with specific antibody at 2h p.i. Bars = 10 μm

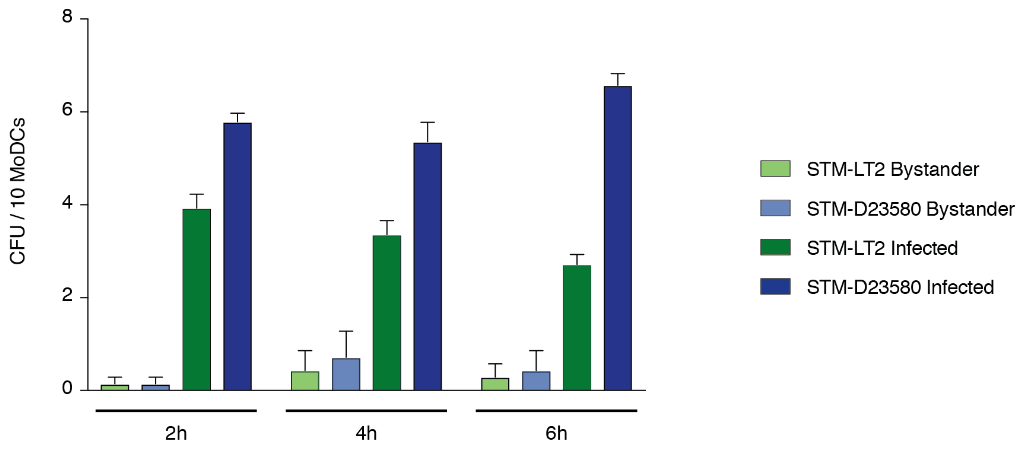

Supplementary Figure 4. Bacterial presence in infected and bystander cells.

CFU enumerated from fluorescently labelled MoDCs. Bystander cells have no or few surviving bacteria. Infected cells contain constant numbers of live bacteria over time. Mean ± SEM from three independent experiments are shown.

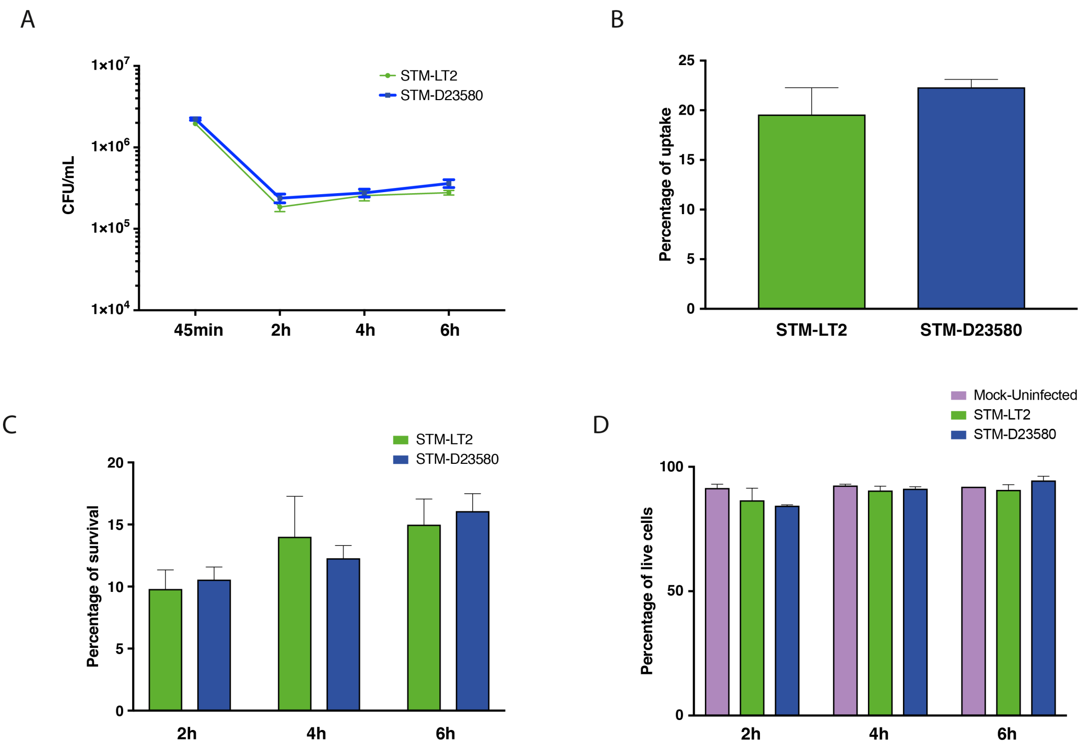

Supplementary Figure 5. Intracellular growth, uptake and survival of bacteria and MoDC viability following STM-LT2 or STM-D23580 treatment.

A No differences were observed in the intracellular growth of STM-LT2 and STM-D23580. The mean ± SEM from three independent experiments is shown. Two-way ANOVA test. **B** Bacteria uptake, calculated as the number of bacteria recovered at 45 min p.i. divided by the initial number of bacteria, didn't show any significant difference. The mean ± SEM from three independent experiments is shown. A two-tailed paired Student *t*-test was used. **C** The percentage of bacterial survival calculated as the number of bacteria recovered at each time point p.i. divided by the number of bacteria recovered at 75min p.i., didn't show any significant difference. The mean ± SEM from three independent experiments is shown. Two-way ANOVA test. **D** MoDC viability was not affected by either *Salmonella* strain. The mean ± SEM from three independent experiments is shown. Two-way ANOVA test.

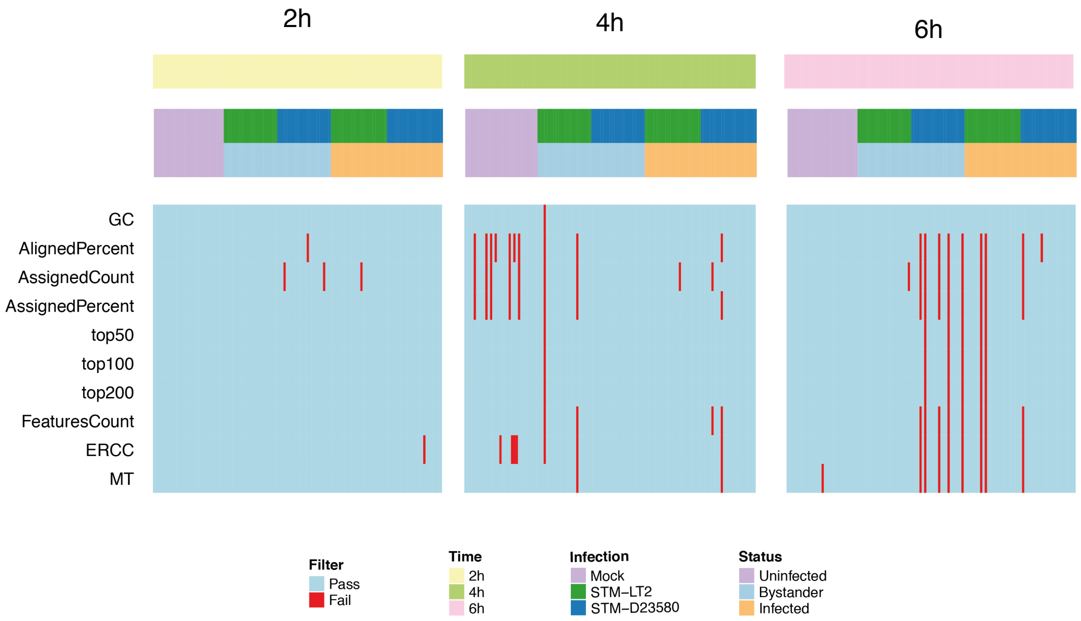

Supplementary Figure 6. Cell filtering quality control.

Outliers were detected across a set of quality control metrics, beyond an appropriate number of median absolute deviations (MADs) above, below, or further than the median value of all single cells, according to the nature and inherent variability of the metric: GC content (±3 MADs), alignment rate (-3 MADs), library size (±3 MADs), percentage reads assigned to genes (-3 MADs), library complexity for the top 50, 100, and 200 genes, respectively (+3 MADs), count of detected genes (-2 MADs), ERCC content (+5 MADs), mitochondrial gene content (+3 MADs). The heat map displays the pass or fail status for each single cell and each quality control filter as blue and red cell, respectively.

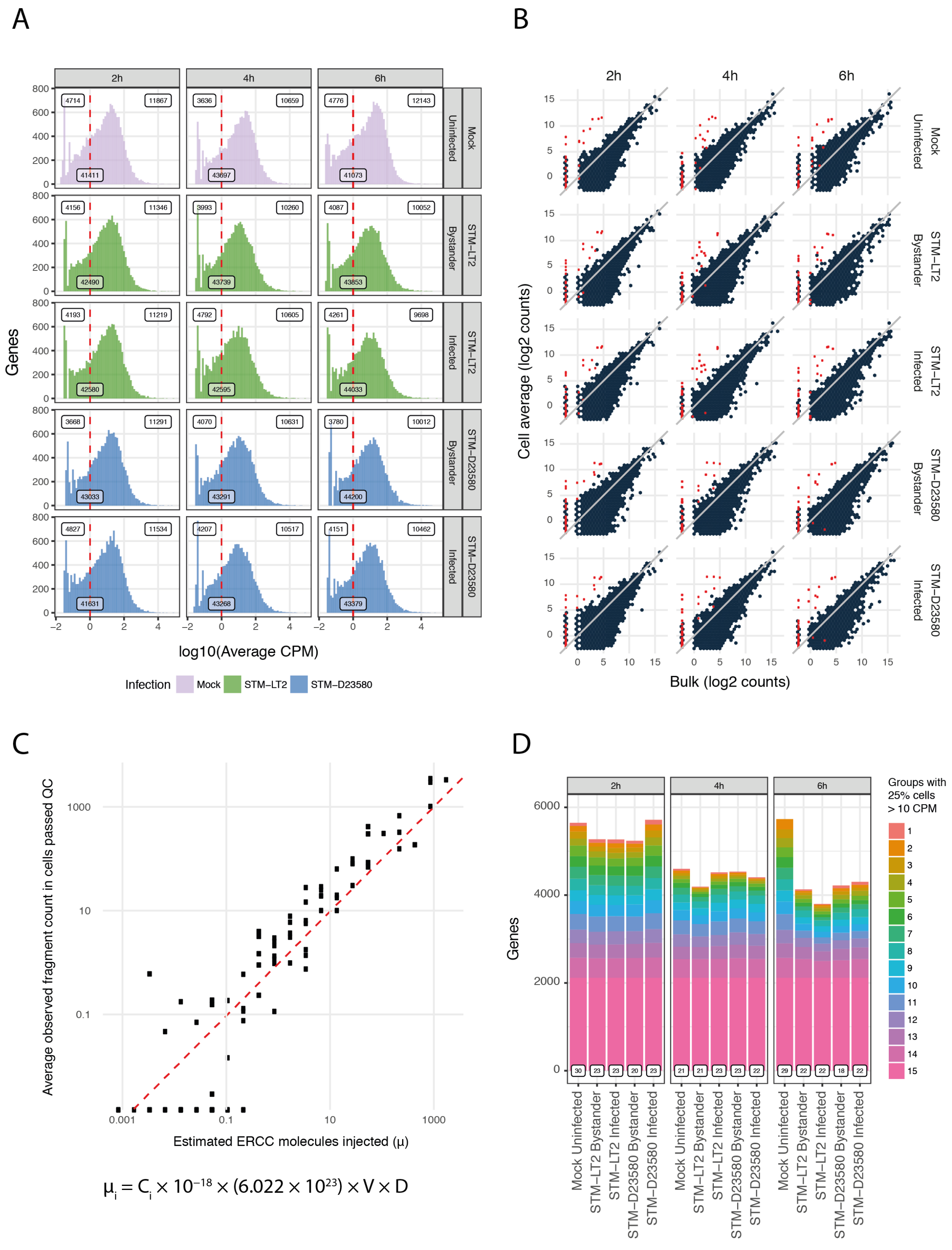
A
B
C
D
2h
4h
6h
Mock
Uninfected
STM–LT2
Bystander
Infected
STM–D23580
Genes
log10(Average CPM)
Infection
Mock
STM–LT2
STM–D23580
Cell average (log2 counts)
Bulk (log2 counts)
Average observed fragment count in cells passed QC
Estimated ERCC molecules injected (μ)
μi = Ci × 10−18 × (6.022 × 1023) × V × D
Groups with
25% cells
> 10 CPM
Mock Uninfected
STM–LT2 Bystander
STM–LT2 Infected
STM–D23580 Bystander
STM–D23580 Infected

Supplementary Figure 7. Gene filtering quality control.

A On average, 10,820 genes were detected above an average 1 CPM (top-right label), and 4,221 genes were detected below an average 1 CPM (top-left label), respectively. Undetected gene counts are shown in the bottom label. **B** Correlation between average log_2-transformed counts in cells from each condition, and matched bulk samples prepared from 5,000 cells sorted from MoDCs mock-infected, challenged with STM-LT2, or challenged with STM-D23580. Red dots indicate ERCC spike-in molecules. **C** Correlation between the average count of fragments yielded for each ERCC spike-in plotted against the estimated count of molecules injected in each sample. The input number of each spike-in molecule per cell μ_i was estimated using the formula shown at the bottom (obtained from https://github.com/catavallejos/TutorialBASiCS). Symbols: C_i: concentration of the spike i in the ERCC mix; V: volume added (10μL); D: dilution factor (1:10,000,000). **D** Stacked bar plot indicating genes detected in each experimental group (>25% of cells above 10 CPM), the total count of experimental groups in which those genes were detected (maximum: 15). Overall, 8,206 appreciably expressed genes were retained for downstream analyses (> 10 CPM in at ≥ 25% of cells of ≥ 1 experimental condition). The count of cells that passed quality filtering within each experiment group is indicated by a label at the bottom of each bar.

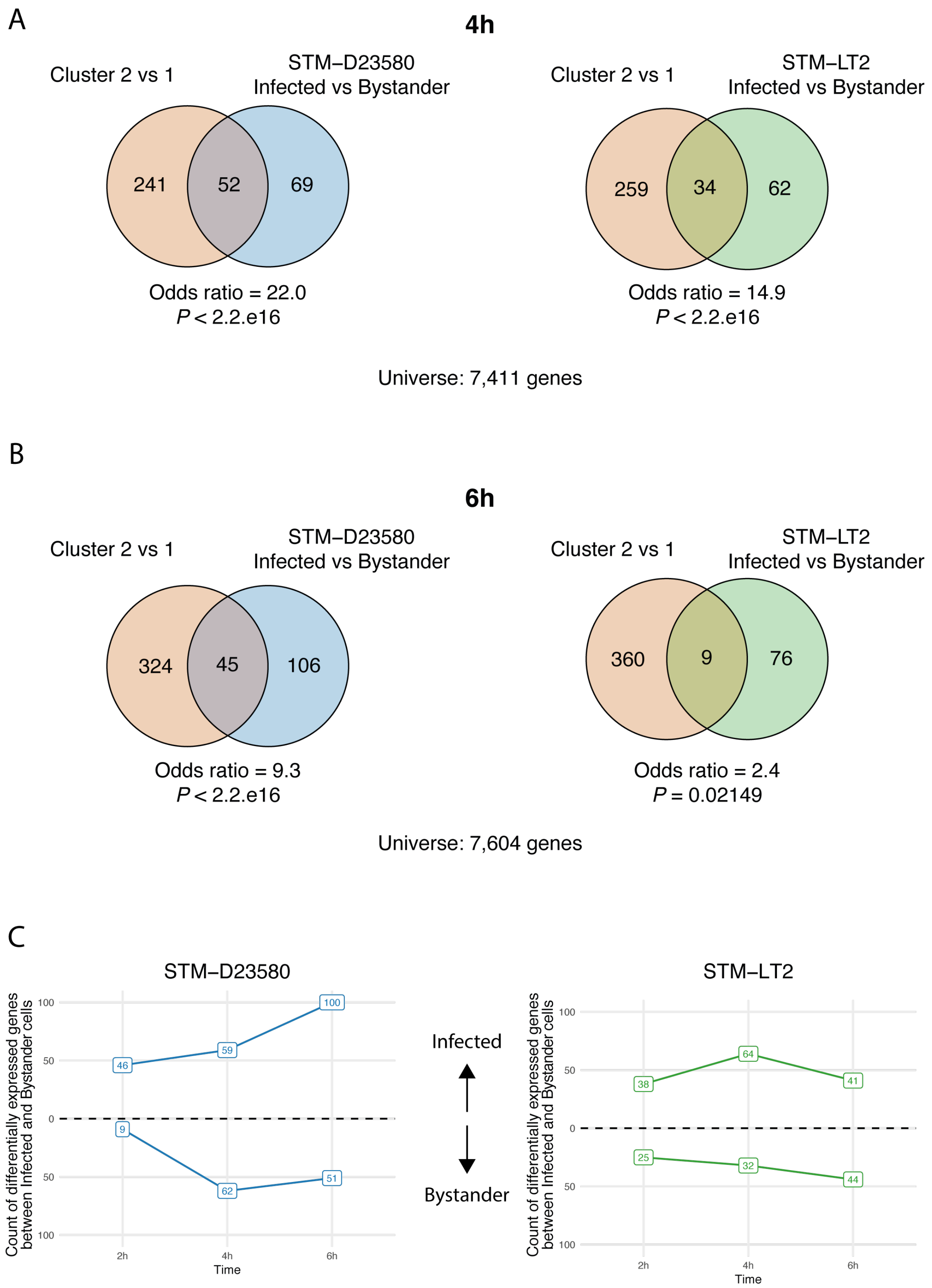
A
4h
Cluster 2 vs 1
STM–D23580
Infected vs Bystander
241
52
69
Odds ratio = 22.0
P < 2.2.e16
Cluster 2 vs 1
STM–LT2
Infected vs Bystander
259
34
62
Odds ratio = 14.9
P < 2.2.e16
Universe: 7,411 genes
B
6h
Cluster 2 vs 1
STM–D23580
Infected vs Bystander
324
45
106
Odds ratio = 9.3
P < 2.2.e16
Cluster 2 vs 1
STM–LT2
Infected vs Bystander
360
9
76
Odds ratio = 2.4
P = 0.02149
Universe: 7,604 genes
C
STM–D23580
Count of differentially expressed genes
between Infected and Bystander cells
100
50
0
50
100
46
59
100
9
62
51
2h
4h
6h
Time
Infected
Bystander
STM–LT2
Count of differentially expressed genes
between Infected and Bystander cells
100
50
0
50
100
38
64
41
25
32
44
2h
4h
6h
Time

Supplementary Figure 8. Differential expression between unsupervised clusters, and between infected and bystander MoDCs.

A, B The differentially expressed (DE) genes (*P*-value < 0.01) identified between cluster 2 and cluster 1 at 4h p.i. and 6h p.i. were compared with the DE genes identified between infected and bystander MoDCs challenged with STM-D23580 or STM-LT2. Venn diagrams are accompanied by the observed odds ratio (OR), the nominal *P*-value of Fisher's Exact Test, and the total count of genes detected at that time point, used as background (i.e., universe) for the test. **A** At 4h p.i., the DE genes identified between unsupervised clusters significantly overlap with the DE genes identified between infected and bystander MoDCs, in both STM-D23580 and STM-LT2 infection models. **B** At 6h p.i., the DE genes identified between unsupervised clusters continue to significantly overlap with the DE genes identified between infected and bystander MoDCs challenged with STM-D23580, but only display a weak enrichment (OR = 2.4; *P*-value = 0.02) for the DE genes identified between infected and bystander MoDCs challenged with STM-LT2. **C** Count of DE genes identified between infected and bystander MoDCs challenged with STM-D23580 and STM-LT2 at each time point. Count of genes significantly up-regulated in the infected and bystander MoDCs are indicated above and below the *x*-axis, respectively.

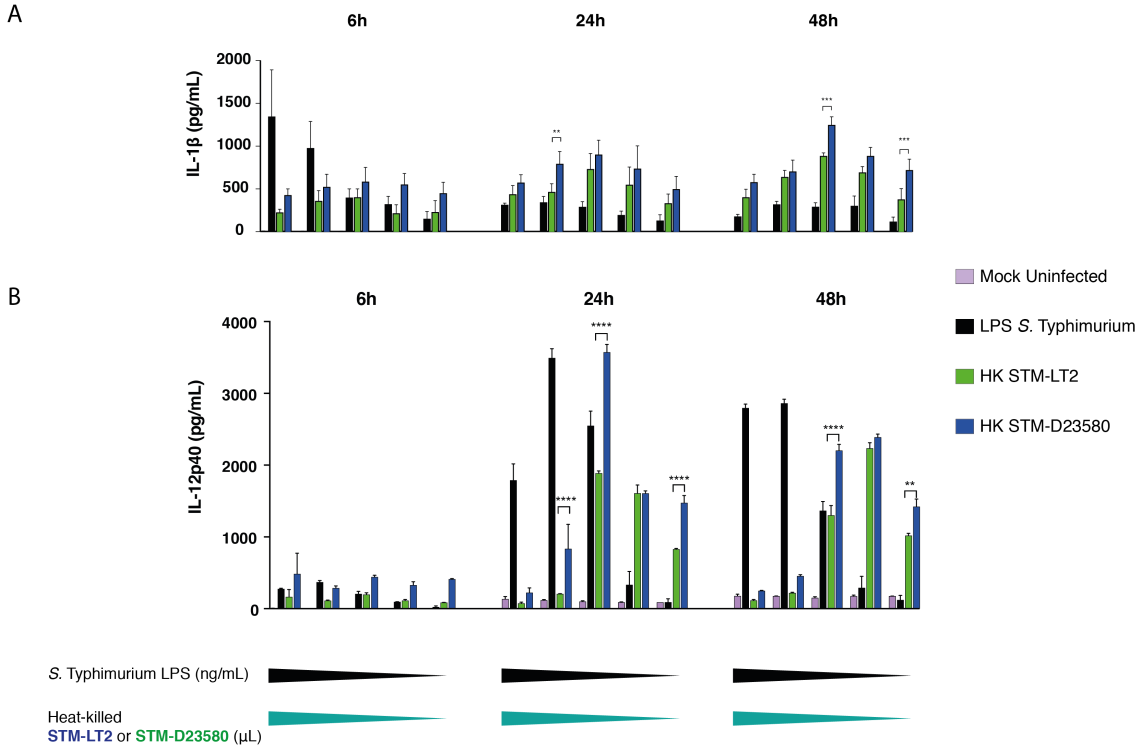

Supplementary Figure 9. IL1β and IL12-p40 secretion following exposure to heat-killed *Salmonella*

DNase and Proteinase treated Heat-killed (HK) bacteria were used in different amounts (50uL-1uL) to stimulate MoDCs. IL-1β (**A**) and IL12-p40 (**B**) were quantified by ELISA at 6h, 24h and 48h p.i. The mean ± SEM from three independent experiments is shown. Two-way ANOVA test, *P*-value < 0.01 (**), < 0.001(***), < 0.0001(****).

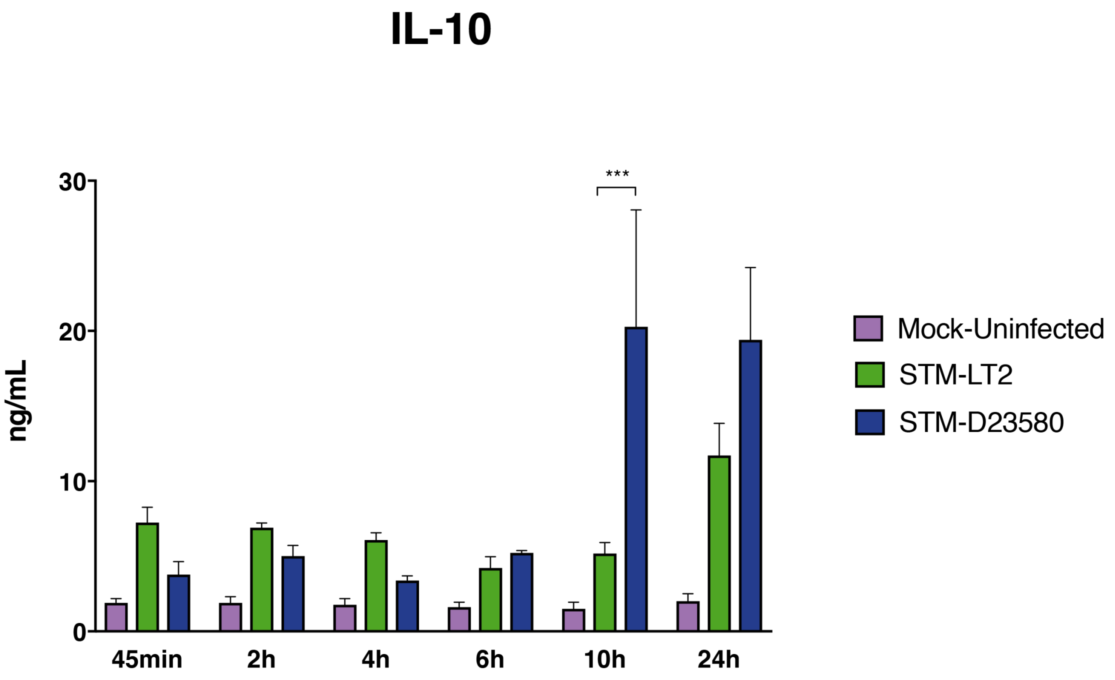

Supplementary Figure 10. IL-10 secretion following exposure to *Salmonella* strains.

IL-10 was quantified by ELISA at 45min, 2h, 4h, 6h, 10h and 24h p.i. The mean ± SEM from three independent experiments is shown. Two-way ANOVA test, *P*-value < 0.001(***).

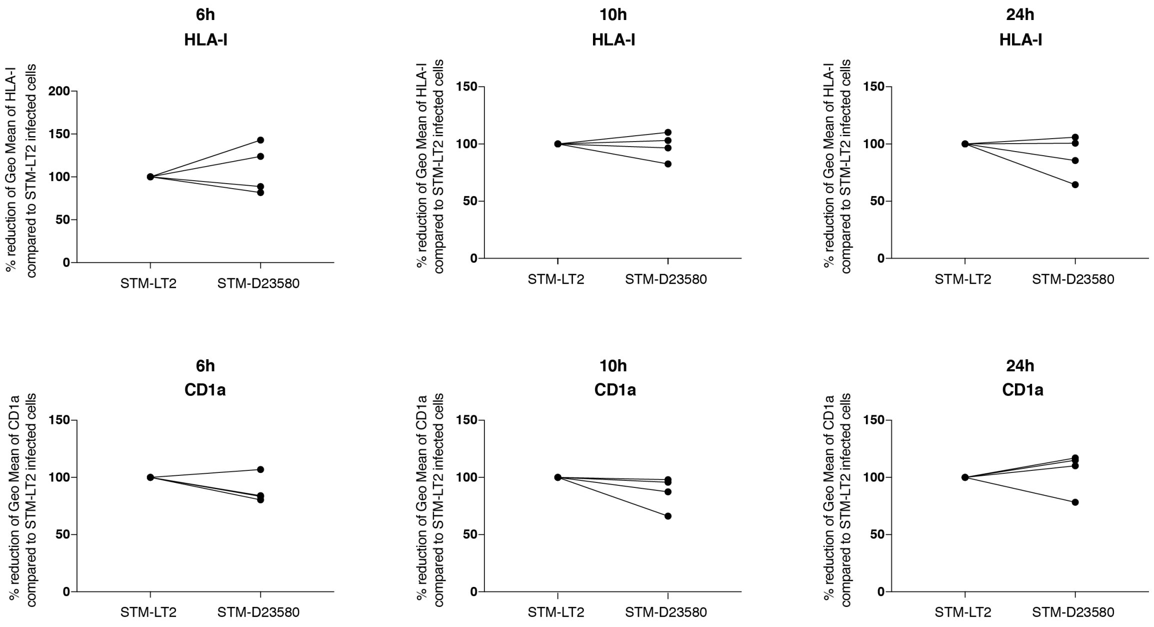

Supplementary Figure 11. STM-D23580 does not affect HLA-I or CD1a expression.

Geometric mean of fluorescence intensity for HLA-I and CD1a on STM-D23580 infected cells plotted as a percentage of the level in STM-LT2 infected cells, set at 100%. Four donors are shown. A two-tailed paired Student *t*-test was used.

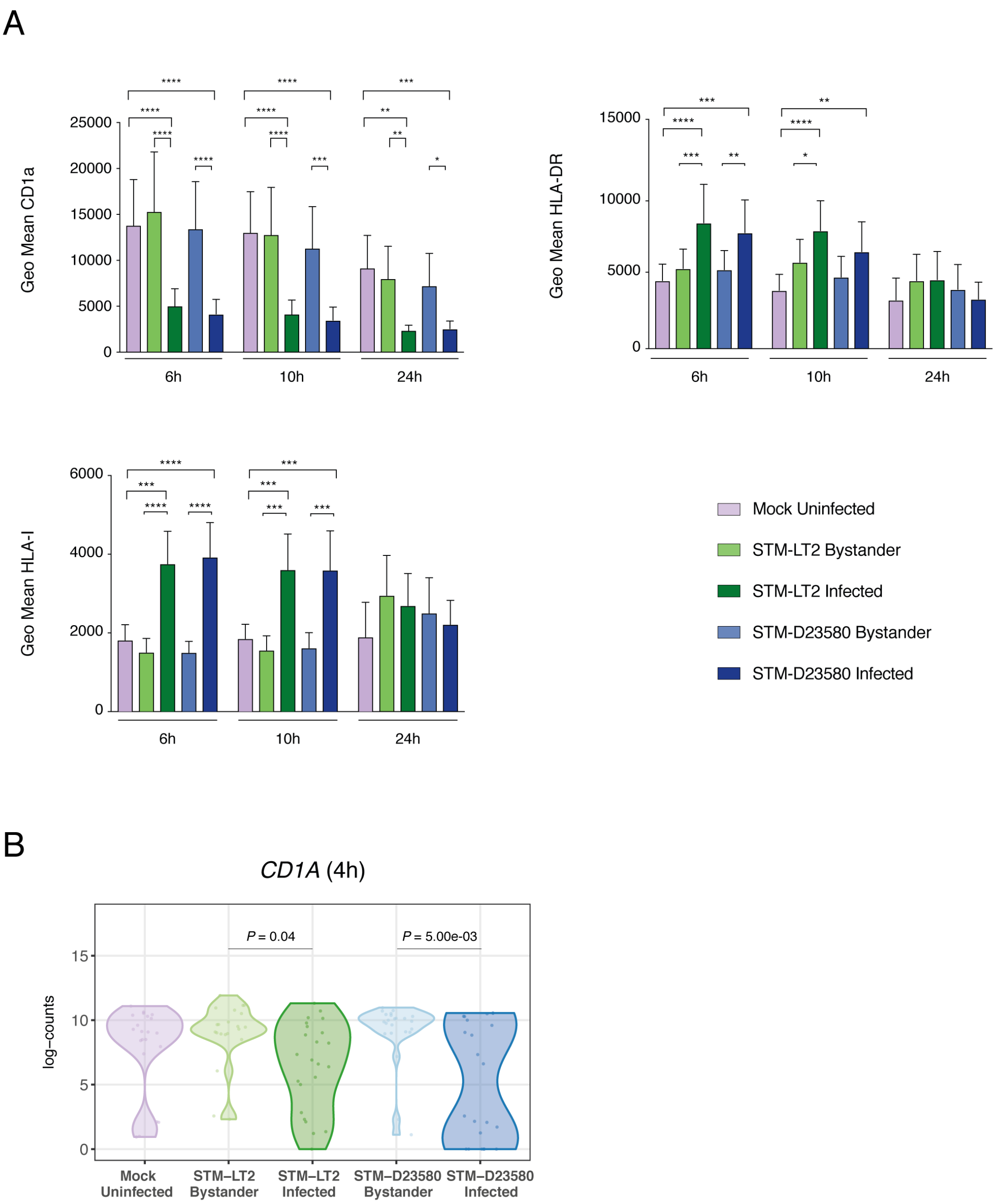

Supplementary Figure 12. Reduced *CD1A* expression in infected cells.

A Histograms showing the surface expression of CD1a, HLA-DR and HLA-I measured by FACS at 6h, 10h, or 24h p.i. in all experimental groups. The mean ± SEM from four independent experiments is shown. Two-way ANOVA, *P*-value <0.05 (*), <0.01 (**), <0.001 (***), < 0.0001 (****). **B** Violin plot showing single-cell gene expression of *CD1A* at 4h p.i. in all experimental groups. Empirical *P*-values were computed from the *scde* Z score using the *pnorm* function of the R *stats* package.

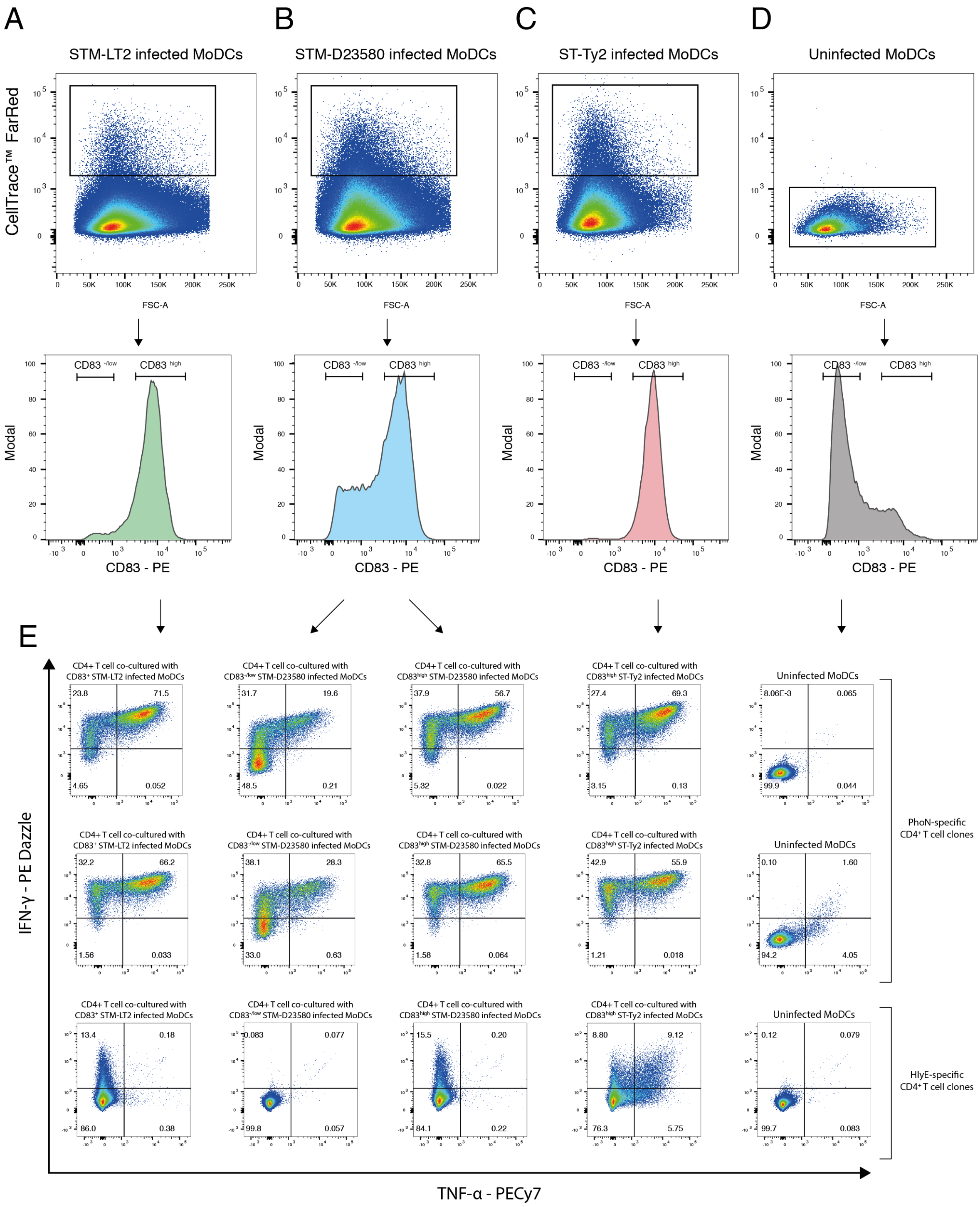
A
B
C
D
STM-LT2 infected MoDCs
STM-D23580 infected MoDCs
ST-Ty2 infected MoDCs
Uninfected MoDCs
CellTrace™ FarRed
FSC-A
Modal
CD83 -/low
CD83 high
CD83 - PE
E
CD4+ T cell co-cultured with CD83+ STM-LT2 infected MoDCs
CD4+ T cell co-cultured with CD83-/low STM-D23580 infected MoDCs
CD4+ T cell co-cultured with CD83high STM-D23580 infected MoDCs
CD4+ T cell co-cultured with CD83high ST-Ty2 infected MoDCs
Uninfected MoDCs
23.8
71.5
4.65
0.052
31.7
19.6
48.5
0.21
37.9
56.7
5.32
0.022
27.4
69.3
3.15
0.13
8.06E-3
0.065
99.9
0.044
32.2
66.2
1.56
0.033
38.1
28.3
33.0
0.63
32.8
65.5
1.58
0.064
42.9
55.9
1.21
0.018
0.10
1.60
94.2
4.05
13.4
0.18
86.0
0.38
0.083
0.077
99.8
0.057
15.5
0.20
84.1
0.22
8.80
9.12
76.3
5.75
0.12
0.079
99.7
0.083
PhoN-specific CD4+ T cell clones
HlyE-specific CD4+ T cell clones
IFN-γ - PE Dazzle
TNF-α - PECy7

Supplementary Figure 13. Flow cytometry strategy demonstrating PhoN and HlyE specific T cell responses to *S.* typhimurium or *S.* typhi infected MoDCs

At 6h p.i., infected cells were sorted based on their CD83 surface expression: **A** STM-LT2 and **C** ST-Ty2 infected MoDCs were sorted as $CD83^{+}$, while **B** STM-D23580 Infected MoDCs were sorted as $CD83^{-/low}$ or $CD83^{high}$. **D** Uninfected MoDCs were used as negative control.

Sorted subsets of infected MoDCs were co-cultured overnight with PhoN or HlyE specific $CD4^{+}$ T cell clones cross-reactive against both Typhoidal and non-Typhoidal serovars; or HlyE specific $CD4^{+}$ T cell clones reactive against Typhoidal serovars.

E $CD4^{+}$ T cells were tested for their ability to secrete IFN-γ and TNF-α, detected by intracellular staining. FACS plots show the percentage of IFN-γ^{+} and/or TNF-α^{+} cells out of the total $CD3^{+}$ $CD4^{+}$ gate. A lower percentage of T cells was activated by $CD83^{-/low}$ STM-D23580 infected MoDCs as compared to $CD83^{high}$ STM-D23580, $CD83^{+}$ STM-LT2 and ST-Ty2 infected cells. HlyE specific $CD4^{+}$ T cell clones were activated only in the presence of $CD83^{+}$ ST-Ty2 infected MoDCs.

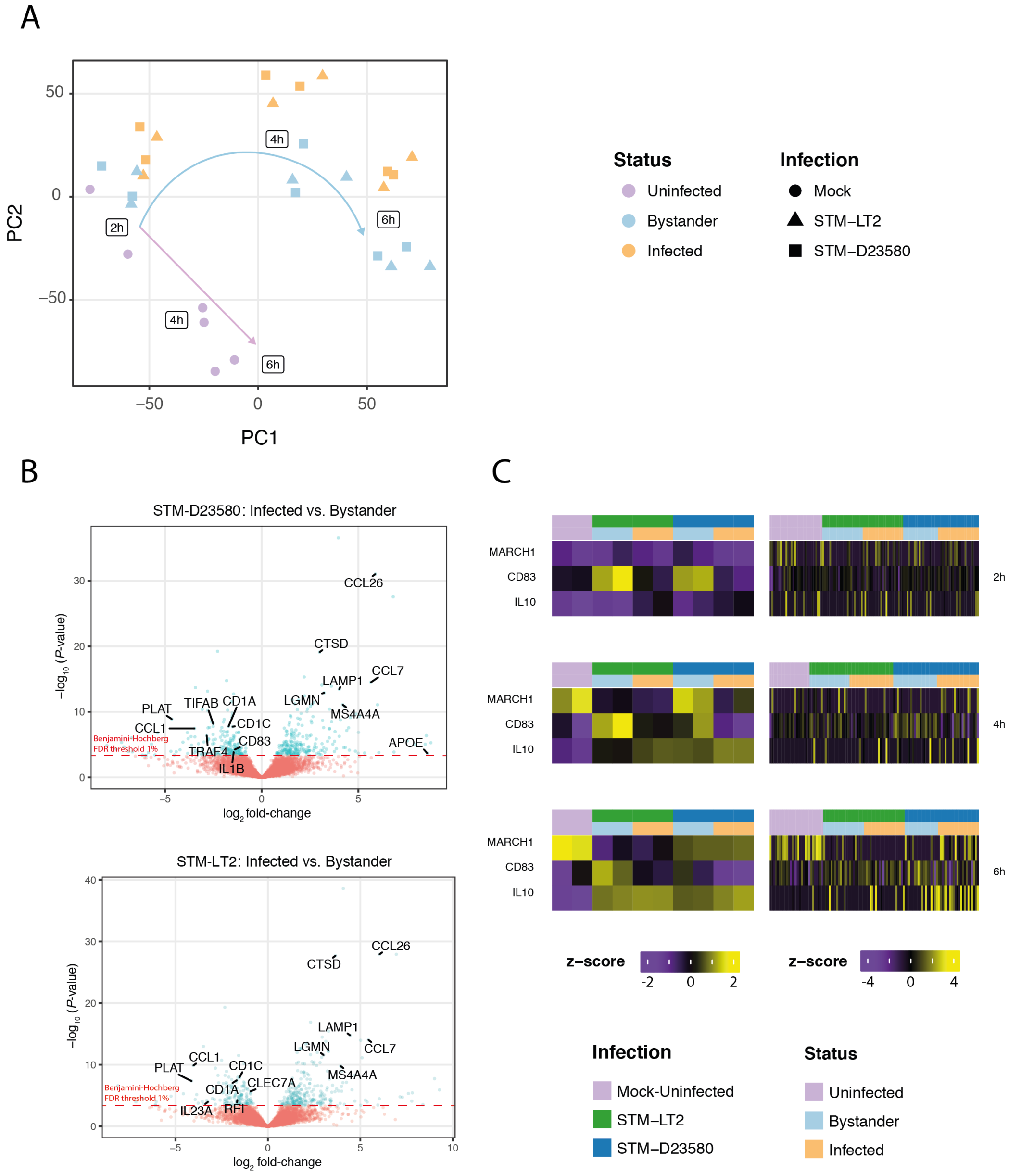

Supplementary Figure 14. Bulk RNA-seq confirms scRNA-seq findings.

A PCA of bulk samples (5,000 cells/sample) showing a distribution of samples similar to the *t*-SNE embedding of single-cell data. **B** Volcano plots showing DE genes (*P*-value < 0.01) between infected and bystander cells in STM-D23580 or STM-LT2 challenged MoDCs identified using the Bioconductor package *DESeq2*. **C** Heat maps displaying *MARCH1*, *CD83* and *IL10* row-scaled normalised expression levels in bulk samples and single cells, respectively.

A

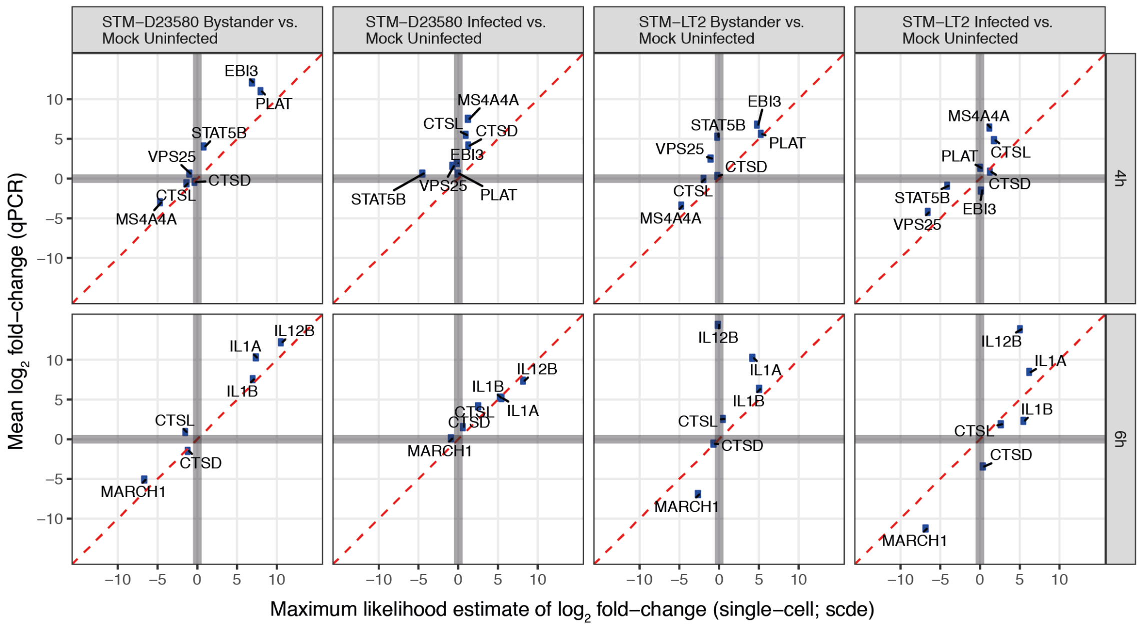

STM−D23580 Bystander vs. Mock Uninfected
STM−D23580 Infected vs. Mock Uninfected
STM−LT2 Bystander vs. Mock Uninfected
STM−LT2 Infected vs. Mock Uninfected
4h
6h
EBI3
PLAT
STAT5B
VPS25
CTSL
CTSD
MS4A4A
IL12B
IL1A
IL1B
MARCH1
Mean log2 fold−change (qPCR)
Maximum likelihood estimate of log2 fold−change (single−cell; scde)

B

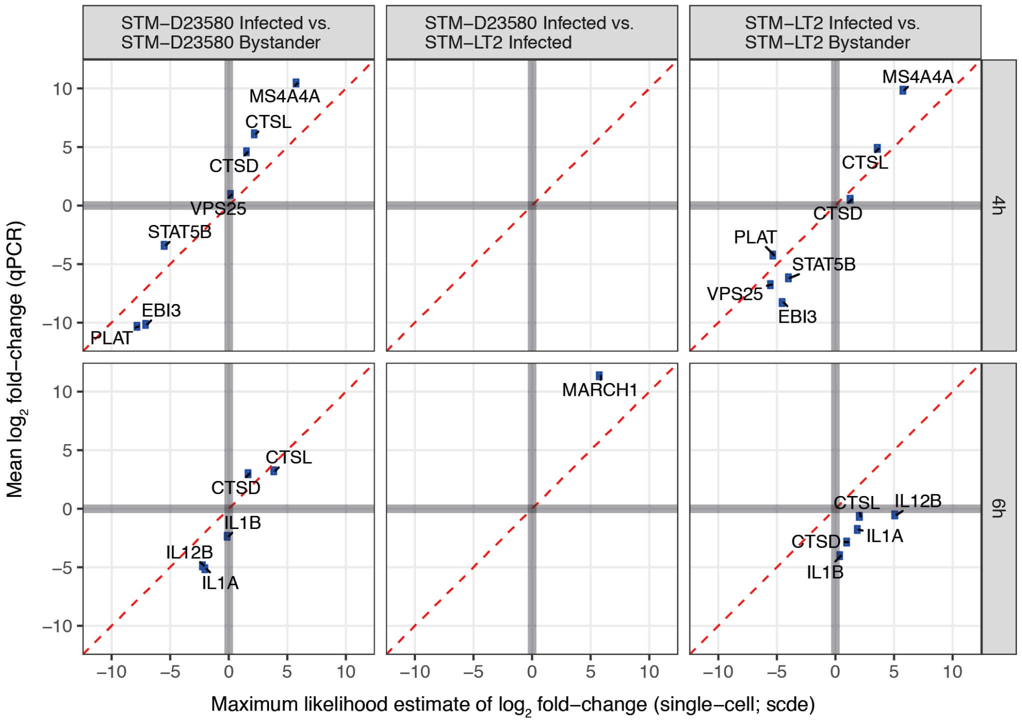

STM−D23580 Infected vs. STM−D23580 Bystander
STM−D23580 Infected vs. STM−LT2 Infected
STM−LT2 Infected vs. STM−LT2 Bystander
4h
6h
MS4A4A
CTSL
CTSD
VPS25
STAT5B
EBI3
PLAT
IL1B
IL12B
IL1A
MARCH1
Mean log2 fold−change (qPCR)
Maximum likelihood estimate of log2 fold−change (single−cell; scde)

Supplementary Figure 15. qPCR data validate scRNA-seq findings.

Mean log fold-change obtained by qPCR ($2^{-\Delta Ct}$; five independent donors) was compared to the maximum likelihood estimate (MLE) of expression log fold-change in single-cell data. **A** Comparisons of *Salmonella*-challenged MoDCs against uninfected MoDCs. Pearson's product-moment correlation coefficient was computed across all panels (r = 0.81; *P*-value = 5.8e-13). **B** Direct comparisons of *Salmonella*-challenged MoDCs. Pearson's product-moment correlation coefficient was computed across all panels (r = 0.88; *P*-value = 7.8e-09).

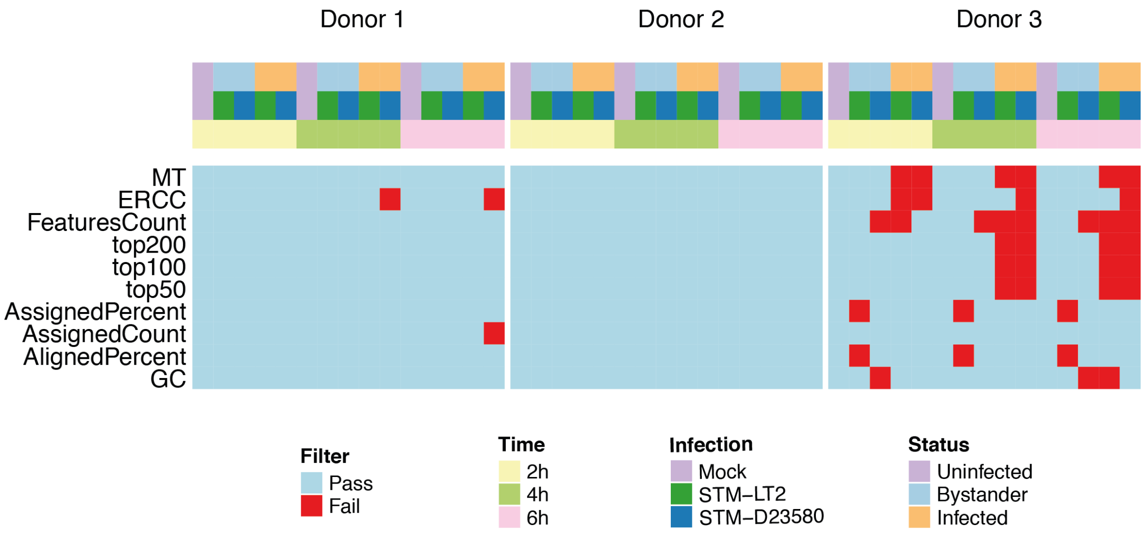

Supplementary Figure 16. Small bulks filtering quality control.

Outliers were detected across a set of quality control metrics, beyond an appropriate number of median absolute deviations (MADs) above, below, or further than the median value of all minibulks, according to the nature and inherent variability of the metric**:** GC content (±3 MADs), alignment rate (-3 MADs), library size (±3 MADs), percentage reads assigned to genes (-3 MADs), library complexity for the top 50, 100, and 200 genes, respectively (+3 MADs), count of detected genes (-2 MADs), ERCC content (+5 MADs), mitochondrial gene content (+3 MADs). The heat map displays the pass or fail status for each minibulk and each quality control filter as blue and red cell, respectively.

Initial number of single cells	Passed	Failed	Infection	Status	Time
**30**	30	0	Mock	Uninfected	2h
**31**	21	10	Mock	Uninfected	4h
**30**	29	1	Mock	Uninfected	6h
**23**	23	0	STM-LT2	Bystander	2h
**23**	21	2	STM-LT2	Bystander	4h
**23**	22	1	STM-LT2	Bystander	6h
**24**	23	1	STM-LT2	Infected	2h
**24**	23	1	STM-LT2	Infected	4h
**24**	22	2	STM-LT2	Infected	6h
**23**	20	3	STM-D23580	Bystander	2h
**23**	23	0	STM-D23580	Bystander	4h
**23**	18	5	STM-D23580	Bystander	6h
**24**	23	1	STM-D23580	Infected	2h
**24**	22	2	STM-D23580	Infected	4h
**24**	22	2	STM-D23580	Infected	6h

Supplementary Table 1: Exclusion of single-cell RNA-sequencing libraries by experimental group. For each experimental group the count of cells that passed quality control, as well as the initial count of cells, are displayed.

Metric	Fail	Pass
**GC**	1	372
**AlignedPercent**	20	353
**AssignedCount**	21	352
**AssignedPercent**	16	357
**top50**	6	367
**top100**	6	367
**top200**	6	367
**FeaturesCount**	12	361
**ERCC**	16	357
**MT**	11	362

Supplementary Table 2: Exclusion of single-cell RNA-sequencing libraries by quality control filters. Count of cells that passed or failed each quality control metric.

	2h				4h			6h		
**Cluster**	**0**	**1**	**2**	**3**	**1**	**2**	**3**	**1**	**2**	**3**
Uninfected	3	14	0	13	0	0	21	0	0	29
STM-LT2 bystander	1	12	8	2	7	14	0	16	6	0
STM-LT2 infected	1	16	5	1	19	4	0	14	8	0
STM-D23580 bystander	0	12	6	2	5	17	1	4	14	0
STM-D23580 infected	1	13	6	3	16	6	0	12	10	0

Supplementary Table 3: Distribution of single cells across unsupervised clusters and experimental groups.

	FORWARD	REVERSE	AMPLICON LENGTH
MS4A4A	TGCTCTGTTGTACCCCTGGT	AAACCTCATTAAGTGGTGTGGG	93
NIT1	TCTTCATGCTGGGCTTCATCA	CCTGGGCTGAGCACAAAGTA	104
CTSD	CAGGGCGAGTACATGATCCC	TTGTAGCCTTTGCCTCCCAG	80
CTSL	GCTAATGACACCGGCTTTGT	TTTCAAATCCGTAGCCAACC	202
EBI3	TCATTGCCACGTACAGGCTC	AGAACAGCTGGACATCCGTG	108
PLAT	CCGGGTGGAATATTGCTGGT	GCTCGCTGCAACTTTTGACA	74
STAT5B	CAGAAGAAGGCAGAGCACCA	AGCGGTCATACGTGTTCTGG	97
VPS25	GAGGATGAGGAGTTCCACGG	ATGATCTCGGCCTTGTGCTC	86
MARCH1	CACTGGGACACTGCGCTTT	TCACAGCAGCGTGTATCTGAG	75
IL12B	CTCCTGGACCACCTCAGTTTG	CTCTTTCAGCTCCAGAGCCC	75
IL1A	CCAGTGCTGCTGAAGGAGAT	GCCGTGAGTTTCCCAGAAGA	87
CLECL1	CGTTTCCACTTCAGAGATCTGTTTC	CCTTCCAGTCTTTGGCAGGA	75
IL1B	ACAGATGAAGTGCTCCTTCCA	GTCGGAGATTCGTAGCTGGAT	73

Supplementary Table 4: List of primers used in the study.
