# Supplementary material for: Invasive Salmonella exploits divergent immune evasion strategies in infected and bystander dendritic cell subsets
Source: Nat Commun. 2018 Nov 19;9:4883. doi: 10.1038/s41467-018-07329-0 (PMC6242960; doi:10.1038/s41467-018-07329-0)
Supplement: Supplementary file 3 — Description of Additional Supplementary Files [file 41467_2018_7329_MOESM3_ESM.pdf]

Description of Additional Supplementary Files

Supplementary Data 1
Description: Description of the single cell RNA-sequencing metadata.

Supplementary Data 2
Description: Tables of Monocle2 differential gene expression analyses. **A** Differential expression results between *Salmonella*-challenged cells collected at 2h and 6h p.i. using the *differentialGeneTest* function. **B** Differential gene expression results for the identification of genes that change as a function of the computed pseudotime using the *differentialGeneTest* function.

Supplementary Data 3
Description: Gene Ontology analysis of the three gene modules identified by pseudotime analysis and k-means clustering.

Supplementary Data 4
Description: Differential expression results using *scde* to identify marker genes for each unsupervised cluster. Marker genes were identified by comparing single cells from each unsupervised cluster compared to all other cells at the same time point.

Supplementary Data 5
Description: Gene Ontology analysis of marker genes identified for each unsupervised cluster.

Supplementary Data 6
Description: Differential expression results using *scde* to directly compare experimental groups of cells.

Supplementary Data 7
Description: Differential expression results using *scde* for genes identified as differentially expressed in either STM-LT2, STM-D23580, or both conditions.

Supplementary Data 8
Description: Gene Ontology analysis of differentially expressed genes identified from direct comparison of experimental groups of cells.

Supplementary Data 9
Description: Differential expression results using *DESeq2* to directly compare experimental groups of small bulks.
